# Development of novel lysosome-related signatures and their potential target drugs based on bulk RNA-seq and scRNA-seq for diabetic foot ulcers

**DOI:** 10.1186/s40246-024-00629-1

**Published:** 2024-06-11

**Authors:** Longhai Tan, Junjun Qu, Junxia Wang

**Affiliations:** 1 Department of Dermatology, Tianjin Beichen Hospital, Tianjin, 300400 China; 2 Zhu Xianyi Memorial Hospital of Tianjin Medical University, Tianjin, 300134 China

**Keywords:** Diabetic foot ulcer, Lysosome-related genes, Artificial neural network, Machine learning, Single-cell sequencing analysis, Targeting drugs

## Abstract

**Background:**

Diabetic foot ulcers (DFU) is the most serious complication of diabetes mellitus, which has become a global health problem due to its high morbidity and disability rates and the poor efficacy of conventional treatments. Thus, it is urgent to identify novel molecular targets to improve the prognosis and reduce disability rate in DFU patients.

**Results:**

In the present study, bulk RNA-seq and scRNA-seq associated with DFU were downloaded from the GEO database. We identified 1393 DFU-related DEGs by differential analysis and WGCNA analysis together, and GO/KEGG analysis showed that these genes were associated with lysosomal and immune/inflammatory responses. Immediately thereafter, we identified CLU, RABGEF1 and ENPEP as DLGs for DFU using three machine learning algorithms (Randomforest, SVM-RFE and LASSO) and validated their diagnostic performance in a validation cohort independent of this study. Subsequently, we constructed a novel artificial neural network model for molecular diagnosis of DFU based on DLGs, and the diagnostic performance in the training and validation cohorts was sound. In single-cell sequencing, the heterogeneous expression of DLGs also provided favorable evidence for them to be potential diagnostic targets. In addition, the results of immune infiltration analysis showed that the abundance of mainstream immune cells, including B/T cells, was down-regulated in DFUs and significantly correlated with the expression of DLGs. Finally, we found latamoxef, parthenolide, meclofenoxate, and lomustine to be promising anti-DFU drugs by targeting DLGs.

**Conclusions:**

CLU, RABGEF1 and ENPEP can be used as novel lysosomal molecular signatures of DFU, and by targeting them, latamoxef, parthenolide, meclofenoxate and lomustine were identified as promising anti-DFU drugs. The present study provides new perspectives for the diagnosis and treatment of DFU and for improving the prognosis of DFU patients.

**Supplementary Information:**

The online version contains supplementary material available at 10.1186/s40246-024-00629-1.

## Introduction

DFU is the most common and serious complication in diabetic patients, which is caused by the interaction of multiple risk factors [1]. With the increasing number of diabetic patients worldwide, the incidence of DFUs is also on the rise [2]. The high incidence and disability of DFUs not only lead to a decline in the quality of patients’ lives, but also cause a heavy burden on healthcare and nursing care [3]. It is estimated that between 9.1 and 26.1 million people develop diabetic foot ulcers worldwide each year, with approximately 17% of these cases eventually requiring amputation [4]. It has been shown that a DFU-related amputation occurs every 20 s in the world [5, 6], and the cost of each amputation can be more than $53,500 [7], which imposes a severe financial, physical, and psychological burden on the patient [8]. Currently, the clinical diagnosis of DFU mainly includes the diagnosis of lower limb vasculopathy and peripheral neuropathy [9, 10]. The diagnosis of lower extremity vasculopathy is based on the following criteria: (1) the diagnosis of diabetes mellitus; (2) clinical manifestations of lower extremity ischemia; (3) auxiliary examinations suggesting lower extremity vasculopathy, with an ABI of < 0.9 at rest, or an ABI of > 0.9 at rest, but with discomfort in the lower extremities during exercise, and a decrease in ABI of 15–20% after a plate exercise test, or with stenosis of blood vessels on imaging. The diagnosis of peripheral neuropathy is based on: (1) abnormal temperature sensation; (2) decreased or lost foot sensation in nylon wire examination; (3) abnormal vibration sensation; (4) loss of ankle reflexes; (5) slowing down of 2 or more items of NCV. If two or more of the above five tests are abnormal, the diagnosis is peripheral neuropathy. The clinical diagnosis of DFU mentioned above is complicated and relies heavily on the experience of clinicians, without effective molecular level diagnostic support, that is, there is a lack of effective biomarkers that can be used for clinical diagnosis of DFU. Additionally, there are still limitations in the multiple treatments for DFU, such as treating lower extremity ischemia and foot infections, surgical debridement, intravenous antibiotics, and reducing the pressure of weight-bearing on the ulcers [11–13]. Despite recent advances in the treatment of DFUs, a large proportion of patients with DFUs still develop chronic wounds due to irreversible processes [14]. Thus, it is urgent to identify novel biomarkers for its diagnosis and treatment.

Lysosomes are signaling pivots and degradation centers in eukaryotic cells that not only play key roles in processes such as senescence, cellular homeostasis and development; they also act as signaling centers for signal transduction, energy and amino acid sensing, and autophagy regulation [15]. Multiple aspects of these functions that converge on the lysosome are able to connect; when these pathways are unregulated, they become the basis for a wide range of human diseases [16]. It has been shown that relevant lysosomal dysfunction has been found in a variety of diseases including autoimmune, metabolic and renal disorders such as Parkinson’s disease, diabetes mellitus and lysosomal storage disease [17]. Of these, diabetes is one of the leading causes of chronic wound healing problems. When diabetics develop ulcers, they are at high risk for major complications such as infection and amputation [18]. And the DFU is one of the serious complications of diabetes that leads to chronic wound healing problems. Previous studies have shown that lysosomal abnormalities can cause diabetes mellitus, but DFU, as the most common and serious complication of diabetes mellitus, lysosomal abnormalities also have a contributing role in the development of DFU [17]. Thus, in this direction, it is necessary and clinically significant to identify lysosome-related genes that play critical regulatory roles in DFU by whole transcriptome analysis, to explore the molecular changes and functions of lysosome-related genes in the development of DFU, and to ascertain the molecular mechanisms by which lysosome-related genes contribute to the development of DFU.

In the present study, we found that Clusterin (CLU), glutamyl aminopeptidase (ENPEP) and RAB guanine nucleotide exchange factor 1 (RABGEF1) can be indicated by multiple bioinformatic and machine learning algorithms as novel hub lysosome-related genes of DFU (DLGs). Then we constructed a novel artificial neural network (ANN) diagnostic model based on the DLGs to assist in the clinical diagnosis of DFU at the molecular level, and the constructed ANN model showed good diagnostic performance in the training and validation cohorts. Following that, we explored the immune microenvironment in DFU and its relationship with DLGs through immune infiltration analysis performed by the ssGSEA algorithm. Immediately after that, we performed single-cell transcriptome analysis on DFU patients, which revealed that DLGs were also heterogeneously expressed among different cell types, which provided stronger evidence for DLGs as diagnostic target for DFU. Finally, we used molecular docking method to identify drugs that have potential therapeutic or palliative effects on DFU by targeting DLGs. In conclusion, the present study provides new perspectives for understanding the molecular mechanism of DFU and diagnosis and treatment of DFU.

## Methods

### Data downloading and processing

We downloaded bulk RNA-seq datasets associated with DFU from the GEO database, and datasets that matched the following criteria were included in the present study: Firstly, the dataset is expected to contain unbiased gene expression data, complete annotation information. Second, the DFU patients incorporated into the training cohort and validation cohort should be distinct and independent. Based on the mentioned above criteria, four datasets (GSE80178, GSE134431, GSE7014 and GSE68183) were included in the present study. The basic information of the datasets used in the present study is provided in Table 1. In the present study, GSE80178 and GSE134431 are used as the training cohorts, and GSE7014 and GSE68183 are used as the validation cohorts. Meanwhile, in order to remove the batch effect between datasets caused by different platforms, we used “ComBat” in the R package “sva” to remove the batch effect in the training cohort after merging GSE80178 and GSE134431 and in the validation cohort after merging GSE7014 and GSE68183, respectively [19], and we used principal component analysis (PCA) to assess the effect of de-batching effects. Additionally, the scRNA-seq dataset associated with DFU was obtained from the previous study by *Theocharidis, Georgios et al.* [20].

**Table 1:** The basic information regarding the dataset in the present study

Dataset type	Dataset source	Annotation platform	Ctrl samples	DFU samples
Bulk RNA-seq	GSE134431	GPL18573	8	13
	GSE80178	GPL16686	3	6
	GSE7014	GPL570	6	20
	GSE68183	GPL16686	3	3
scRNA-seq	*Theocharidis, Georgios et al.*	-	10	13

### Differential analysis of gene expression

The expression profiles of DFUs and Ctrls were compared using the R package “limma”, and dysregulated expression genes (DEGs) were identified in both clusters using a P-value < 0.05 as a criterion.

### Weighted correlation network analysis

The “WGCNA” software package was used to identify DFU-related genes in the training cohort [21]. Initially, clustering of samples is performed to detect and exclude outliers to ensure the network analysis is robust. Then constructed the network using a soft thresholding capability to highlight strong correlations and punish weak correlations. Subsequently, we transformed the neighbor-joining matrix into a topological overlap matrix (TOM). Based on the measure of variance of the TOM, and set the minimum module size to 500, and grouped genes with similar expression patterns into the same module by means of average correlation hierarchical grouping. Eventually, we assessed module characterized genes for their correlation with DFUs and identified the matches to the study objectives based on the extent of the correlation.

### Protein-protein interaction analysis

We analyzed protein-protein interactions (PPI) between dysregulated lysosomal genes using the STRING database (https://string-db.org/) and Cytoscape software [22]. Dysregulated lysosomal genes were included in the PPI network, and the CytoHubba function in Cytoscape was utilized to provide a composite score for dysregulated lysosomal genes, and the top 40 genes in the composite score were ultimately selected for subsequent analysis.

### Functional enrichment analysis

Aiming to ascertain which biological processes and functions the top 40 scoring genes were mainly enriched, we performed Gene Ontology (GO) and Kyoto Encyclopedia of Genes and Genomes (KEGG) analysis on the top 40 scoring genes using the R package “clusterProfler” [23]. Pvalue < 0.05 was regarded as statistically significant.

### Selection of hub lysosome-related genes of DFU

We used 3 machine algorithms to identify DLGs, namely: least absolute shrinkage and selection operator (LASSO), support vector machine recursive feature elimination (SVM-RFE), and random forest(RF). It has been shown that machine learning has been extensively applied in the biomedical sciences and is capable of efficiently and rapidly analyzing biological data and accurately identifying hub genes in gene expression profiles [24]. Firstly, we screened the top 40 scored genes to identify potential candidate genes using the “randomForest” package’s RF algorithm, the “glmnet” package’s LASSO algorithm and the “e1071” package’s SVM-RFE algorithm [25–27]. Eventually, we identified 3 overlapping hub lysosome-related genes of DFU (DLGs) by using the upset diagrams to crossover the candidate genes screened by the 3 algorithms mentioned above.

### Diagnostic evaluation of DLGs for DFU

We further investigated whether the selected DLGs had potential value in diagnosing DFU, and thus evaluated the performance of the DLGs. We performed receiver operating characteristic (ROC) analysis using the R software package “pROC” to derive area under roc curve (AUC) values. Specifically, we obtained expression of DLGs and disease state grouping data from DFU sets, performed ROC analysis using the “roc” function of the “pROC” software package, and derived the final AUC results using the “ci” function of “pROC”.

### Construction and verification of the artificial neural network model

We constructed an ANN diagnostic model based on the transcriptome level of DLGs using the R package “neuralnet”. The seed was set to 123 after we normalized the gene expression data using the min-max normalization method.

The constructed ANN model consisted of predominantly three layers:

1) Input layer, which mainly consists of the gene expression of the three normalized DLGs.

2) Hidden layer, which mainly includes the gene expressions of the three normalized DLGs and the weights of the three DLGs, as well as the weights between the three hidden layers.

3) Output layer, which indicates the result of judging whether the sample belongs to the Ctrls or the DFUs.

### Single-gene GSEA analysis

We clustered DFUs based on the median expression values of DLGs with gene set enrichment analysis (GSEA) for different subgroups to study which biofunctions and signalling pathways associated with DLGs, and considered *P* < 0.05 to be statistically significant.

### Evaluation of immune cell infiltration and their correlation with the DLGs

Infiltration of immune cells was assessed using ssGSEA [28]. Specifically, ssGSEA was performed in R language using the R packages “GSVA” and “GSEABase” and the immunological characteristics of DFU patients were assessed using the ssGSEA algorithm, respectively. We firstly obtained the information of the genes of 28 immune cells gene sets (Table S1) from the TISIDB database (http://cis.hku.hk/TISIDB/), and then performed single-sample gene set enrichment analysis (ssGSEA) and calculated ssGSEA scores. We used the “pheatmap” R package to visualize the infiltration levels of different immune cells under different infiltration algorithms. To assess the differential infiltration abundance of different immune cells between Ctrls and DFUs, we used the Wilcox test for pairwise comparisons. Subsequently, we used the “ggplot” R package to visualize the infiltration levels of different immune cells under different infiltration algorithms. Subsequently, we used the “ggplot” R package to visualize the correlation between immune cell infiltration abundance and DLGs expression.

### scRNA-seq analysis

We downloaded scRNA-seq data of DFU from the previous study by *Theocharidis, Georgios et al.* [20]. , and analyzed them using the R package “Seurat” [29]. Before analyzing the scRNA-seq data, we weeded out low-quality cells using the following method: cells are likely to be under stress when they have the highest percentage of mitochondrial genes of all genes. Thus, cells with more than 25% of mitochondrial genes will be filtered. Since low-mass cells or empty droplets usually contain less genes and bicellular cells contain more genes, low-mass cells were filtered using the criteria nFeature RNA < 500 and nFeature RNA > 5000. The results yielded 71,718 cells and 14,776 genes. Next, we normalized the gene expression of the cells using the “NormalizeData” function and performed PCA using the ElbowPlot function to extract the top 20 principal components (PCs), which were further analyzed using the “FindVariableFeatures” function. For unsupervised and unbiased clustering of cell subpopulations, we used the “FindNeighbors”, “FindClusters” (resolution = 0.5) and “RunUMAP” functions. We then annotated the cell types using known markers [30–32].

### Identification and docking of drugs targeting DLGs

Aiming to identify drugs that can target DLGs, we used the Enrichr platform (https://maayanlab.cloud/Enrichr/) for online identification and analysis. First, we entered DLGs gene symbols in the homepage of the Enrichr platform, and then screened the DSigDB database in the “Disease/Drugs” module to identify drugs that target DLGs, and set *P* < 0.05 as statistically significant. Subsequently, we investigated the binding affinity of the screened drugs to DLGs using molecular docking method (MDM) to identify the most promising drugs. Specifically, the molecular structures of screened drugs were acquired from PubChem database (https://pubchem.ncbi.nlm.nih.gov/). Meanwhile, the 3D coordinates of RABGEF1 (PDB ID, 1TXU; resolution, 2.35Å), CLU (PDB ID, 5JM4; resolution, 2.34 Å) and ENPEP (PDB ID, 4KX7; resolution, 2.15Å) were retrieved from the PDB (https://www.rcsb.org/). We used the AutoDock tool for protein-ligand docking and PyMOL to visualize receptor-ligand interactions.

### Statistical analysis

All statistical analyses and visualizations were performed in R language, and *P* < 0.05 was considered statistically significant.

## Results

### Identification of DEGs associated with DFU by WGCNA analysis

**Fig. 1:**
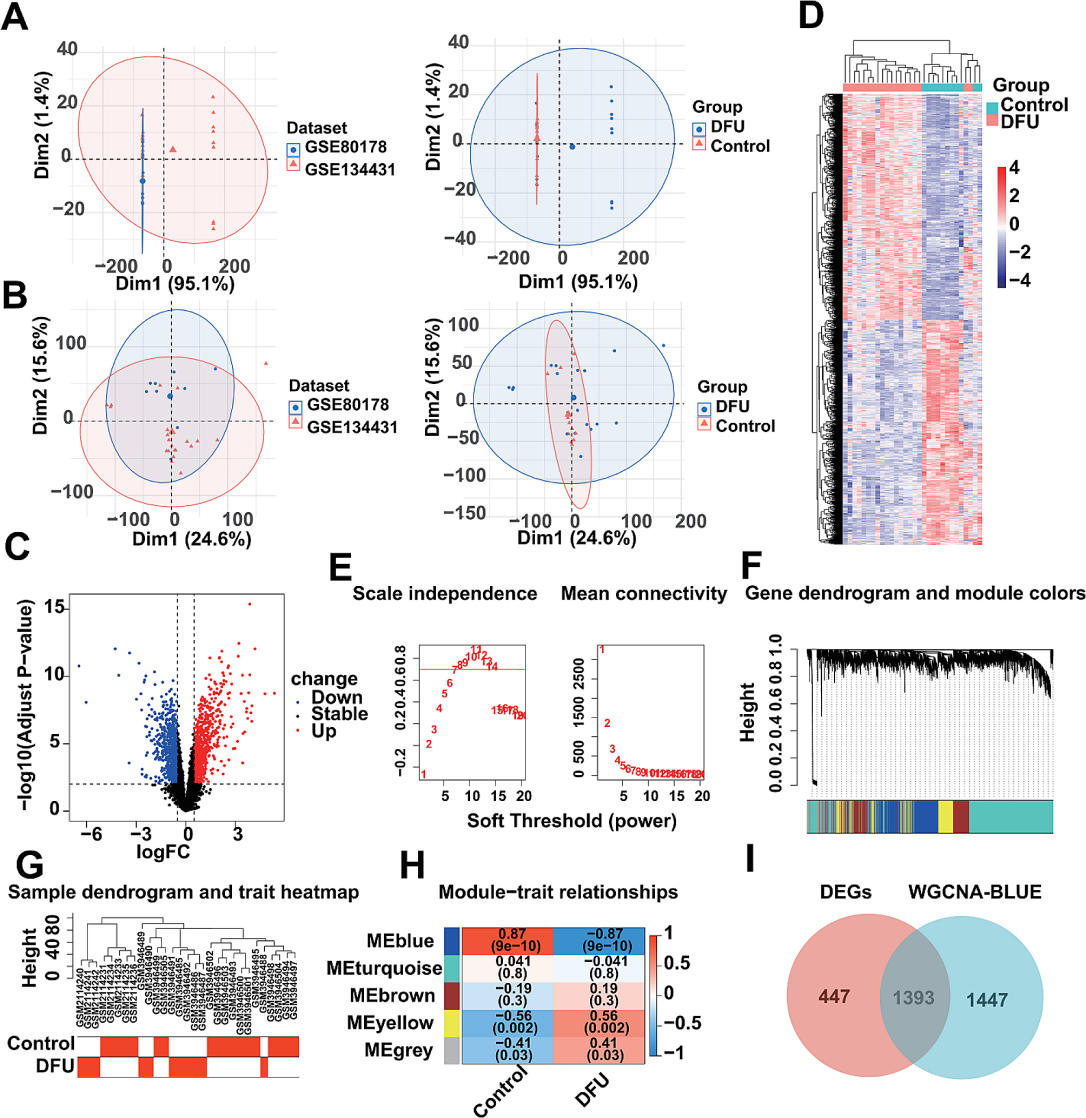
Identification of DFU-related dysregulated expression genes. (**A**) The overall landscape of unprocessed data from the cohort. (**B**) The overall landscape of data from the cohort after removing batch effects. (**C**) Volcano plot showing DEGs, where blue indicates downregulation and red indicates upregulation. (**D**) Heatmap showing the overall landscape of dysregulated expression of DEGs. (**E**) Analysis of network topology for various soft-thresholding powers. (**F**) Clustering dendrogram of genes. (**G**) Clustering dendrogram of DFU and control samples. (**H**) Heatmap of correlation between module genes and phenotypes. (**I**) venn diagram showing overlapped genes between the DEGs and the genes in the blue module

We used GSE80178 and GSE134431 as training cohort for the present study. However, there are a lot of batch effects between different datasets due to different sequencing platforms and personnel differences, thus we eliminated the batch effects in the training cohort. The results after batch removal showed that the samples were evenly dispersed and could be used for subsequent analysis (Fig. 1A-B). Immediately after that, we performed differential gene expression analysis on the training cohort, and obtained 1840 dysregulated expressed genes (DEGs), of which 930 were up-regulated and 910 were down-regulated (Fig. 1C) (Table S2), and the overall landscape of the expression of these DEGs is shown in Fig. 1D. Meanwhile, we performed the WGCNA to identify more DFU-related genes. The scale-free fit exponent and average connectivity analysis for different soft threshold powers are shown in Fig. 1E. The genes were categorized into five independent co-expression modules according to the optimal soft threshold power β = 11 (unscaled R2 = 0.9) (Fig. 1F). The clustering dendrogram showed the clustering characteristics of the samples, and the DFU samples were highly distinguishable from the control samples (Fig. 1G). Correlation plots of module-trait relationships showed that the blue module containing 2840 genes had the highest correlation with DFU (Fig. 1H) (Table S3). Finally, we intersected 1840 DEGs with 2840 DFU-associated genes to obtain 1393 DFU-related DEGs (Fig. 1I).

### Identification of lysosome-related DEGs in DFUs

**Fig. 2:**
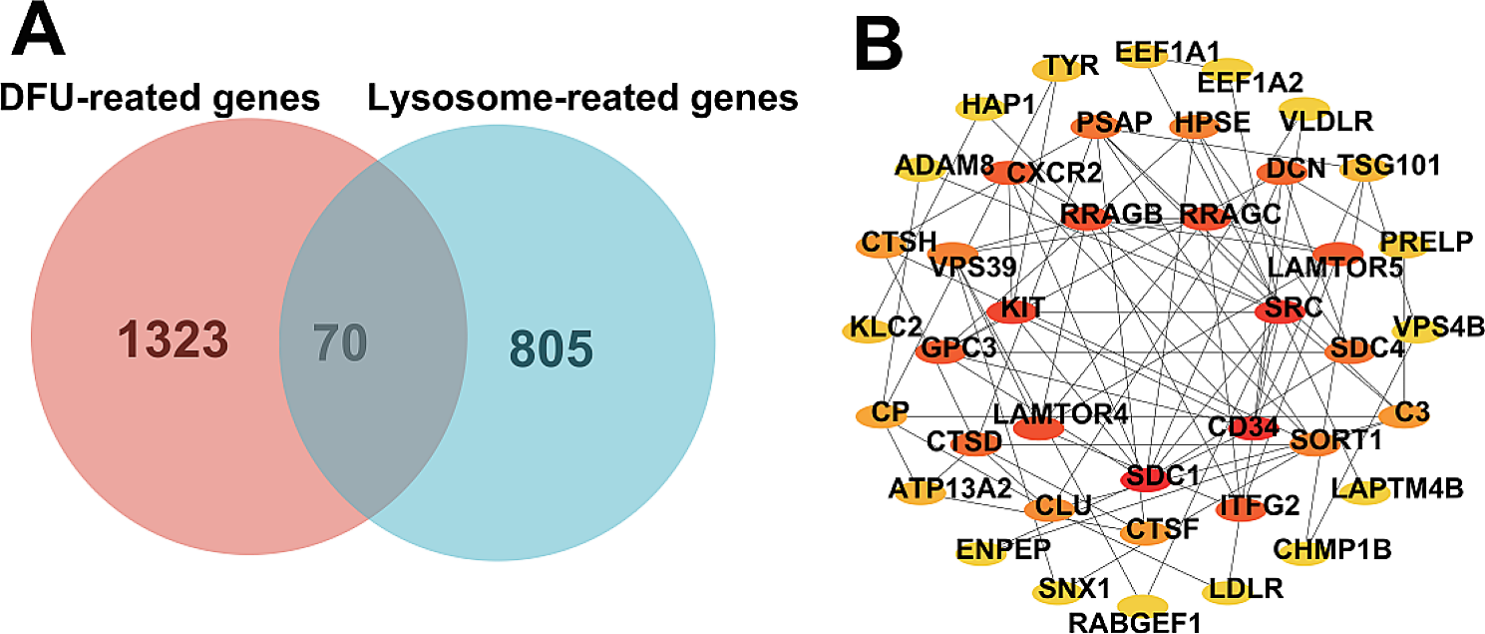
Characterization of lysosomal genes dysregulately expressed in DFU. (**A**) venn diagram showing overlapped genes between the DFU-related DEGs and the lysosome-related genes. (**B**) The protein-protein interaction network showing the interactions between the top 40 scoring genes with the higher scores

Initially, we cross-analyzed the 1393 DFU-related DEGs obtained in the mentioned above results with the 875 lysosome-related genes annotated in AmiGO2 (Fig. 2A). In order to take the key factors of protein interactions into account in the screening process for more accurate results, we included these 70 lysosome-related DEGs in the protein interaction analysis. Specifically, we utilized the STRING database (https://string-db.org/) to construct a protein-protein interactions(PPI) network of these 70 lysosome-related DEGs, and then utilized the CytoHubba plugin in Cytoscape to evaluate these 70 lysosome-related DEGs as a whole and selected the top 40 scoring genes with the highest scores (Fig. 2B) (Table S5). Subsequently, we performed functional enrichment analysis on the top 40 scoring genes identified, in which GO enrichment analysis showed that they were indeed highly related to lysosomes, such as “lysosomal membrane”, “secondary lysosome”, “primary lysosome”, “lysosomal transport” and “lysosomal protein catabolic process”. Also, significantly enriched are pathways related to transmembrane transport, such as “extracellular exosome biogenesis”, “regulation of autophagy”, “regulation of exocytosis”, “transcytosis”, “Phagocytosis”, “pore complex”, “ubiquitin-like protein ligase binding”, “chaperone binding”, “peptide binding” and “ion channel binding”. In addition, they are also associated with immune/inflammatory responses such as “neuroinflammatory response”, “NK T cell differentiation”, “immature B cell differentiation” and “inflammatory cell apoptotic process” (Figure S1 A). The results of KEGG enrichment analyses were similar to those of GO enrichment analyses, showing that they are mainly associated with lysosomes, transmembrane transport of substances, and immune/inflammatory responses such as “Lysosome”, “Endocytosis”, “mTOR signaling pathway”, “Autophagy - animal” and “Cell adhesion molecules” (Figure S1 B). In summary, the pathogenesis of DFUs may be related to the lysosomal pathway and the immune/inflammatory response, which is consistent with previous studies. Autophagy is a lysosome-dependent self-renewal mechanism that degrades and recycles cellular components in eukaryotic cells to maintain the stability of the intracellular environment [33]. It has been previously demonstrated that autophagy plays a crucial role in the various stages of DFU healing, that is, autophagy functioned to contribute to wound healing by regulating the hemostatic/inflammatory, proliferative, and remodeling phases of DFUs [34]. However, dysregulation of autophagy is also an important factor contributing to DFU [35, 36]. It has been demonstrated that advanced glycation end-products (AGEs) can cause refractory wounds by affecting the function of multiple cell types, and that autophagy plays an important role in AGE-induced refractory wounds [37]. A previous study reported that AGEs promote macrophage polarization to the M1 phenotype through autophagy activation, allowing them to kill pathogens early in inflammation and promote the release of inflammatory factors to cause DFU [38, 39].

### Identification of DLGs based on multiple machine learning algorithms

**Fig. 3:**
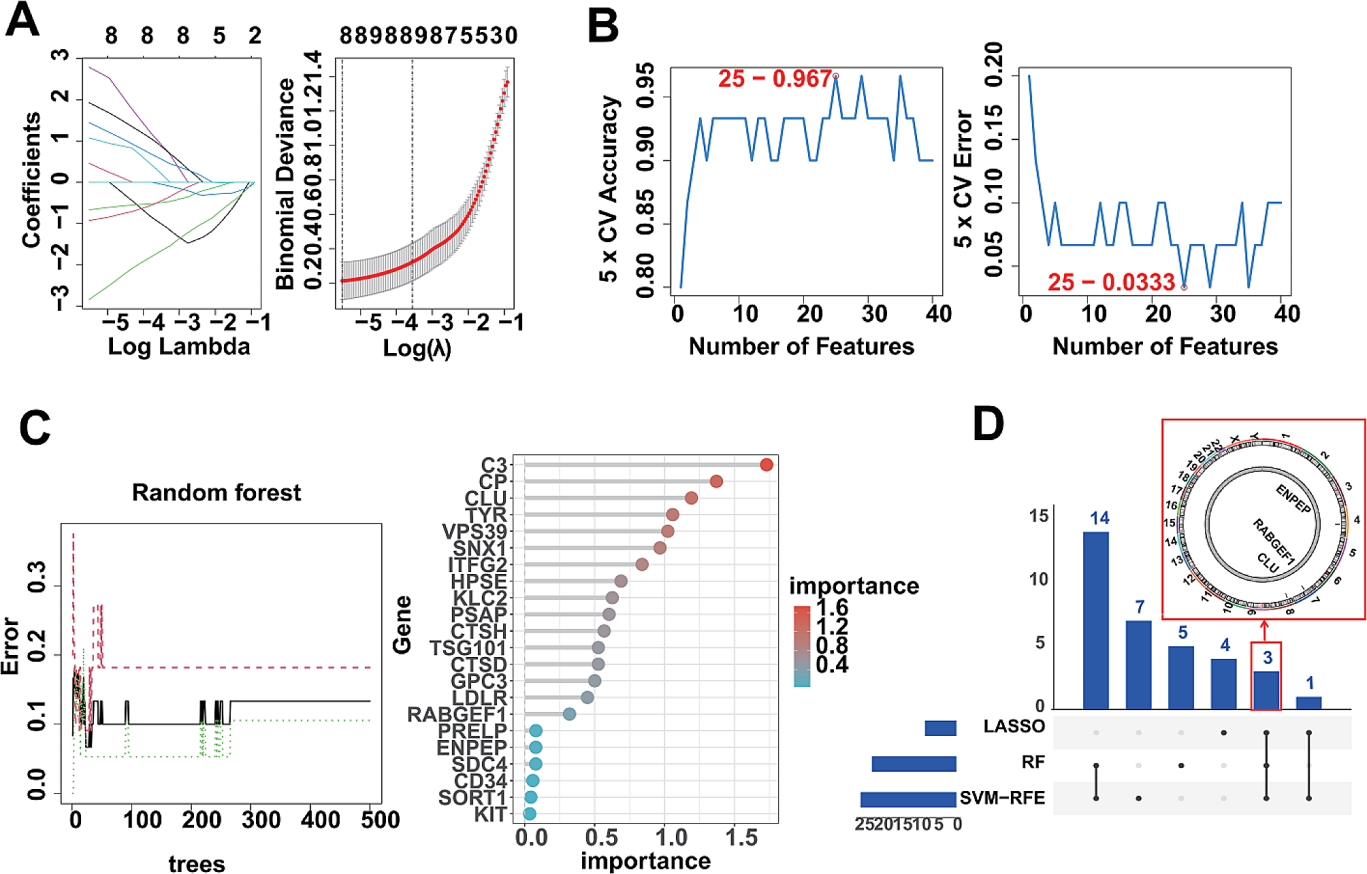
Identification of DLGs using 3 machine learning algorithms. (**A**) LASSO coefficient profiles of the top 40 scoring genes (left panel). After cross-validation for tuning parameter selection, 8 candidate genes were identified (right panel). (**B**) SVM–RFE algorithm identified 25 candidate genes with an accuracy of 0.967 (left panel) and an error of 0.0333 (right panel). (**C**) RandomForest algorithm identified 22 candidate genes. RandomForest error rate versus the number of classification trees (left panel) and gene importance scores (right panel). (**D**) Upset diagram showing the overlapped candidate genes under the three machine learning algorithms

To identify the hub lysosomal genes in DFU, we applied three machine learning algorithms to analyze and identify the top 40 scoring genes, including LASSO, SVM-RFE and RF (Table S6). Firstly, we identified 8 candidate genes using the LASSO algorithm (Fig. 3A), 25 candidate genes using the SVM-RFE algorithm with an accuracy of 0.967 and an error rate of 0.0333 (Fig. 3B), and 22 candidate genes with importance greater than 0 using the RF algorithm (Fig. 3C). Subsequently, we intersected the candidate genes obtained from the mentioned above machine learning algorithms and finally found that CLU, RABGEF1, ENPEP could be indicated by all the algorithms, meaning that CLU, RABGEF1 and ENPEP could be used as DLGs for DFU under the multiplex algorithm (Fig. 3D).

### Evaluation of the diagnostic performance of DLG applying to DFU

**Fig. 4:**
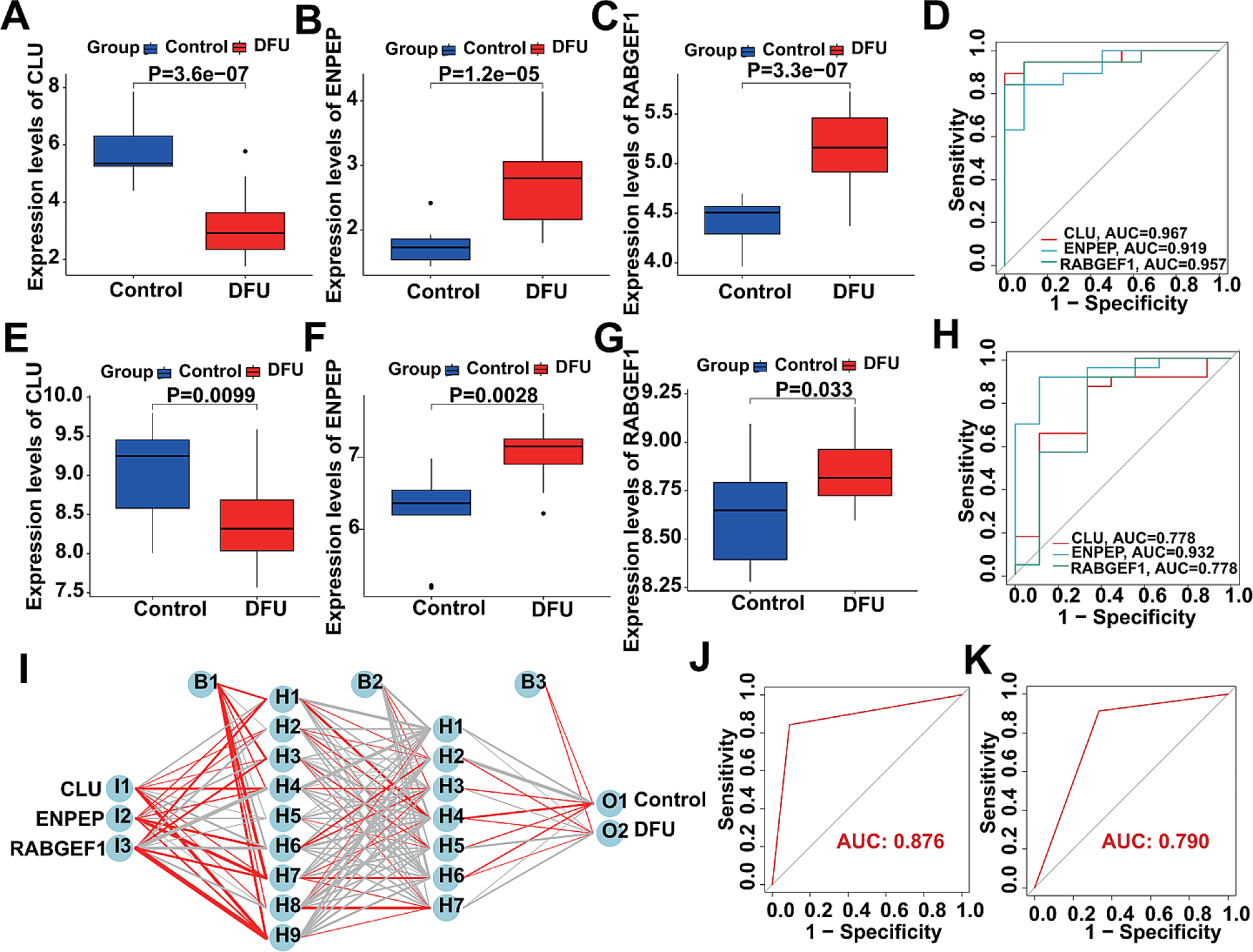
The performance of the screened DLGs. (**A**-**C**). The expression of the screened DLGs between the DFU and Control samples in the training cohort (Wilcoxon test). (**D**) ROC showing the diagnostic performance of the screened DLGs in the training cohort. (**E**-**G**) The expression of the screened DLGs between the DFU and Control samples in the validation cohort (Wilcoxon test). (**H**) ROC showing the diagnostic performance of the screened DLGs in the validation cohort. (**I**) Construction of ANN based on transcriptome expression patterns of CLU, RABGEF1 and ENPEP. (**J**) The AUC of the training set with a value of 0.876. (**K**) The AUC of the validation set with a value of 0.790

The screened DLGs from the training cohort (GSE80178 and GSE134431) were differentially expressed in DFU than those in control (Fig. 4A–C), and the DLGs’ AUCs of the ROC curve were 0.967 of CLU, 0.919 of ENPEP, 0.957 of RABGEF1, respectively (Fig. 4D). Meanwhile, we merged dataset GSE7014 and dataset GSE68183 as the validation cohort of the present study to ensure the reliability of the analysis results. The results showed that DLGs were all deregulated expression in DFU patients, and the deregulation trend was consistent with the training cohort of the present study (Fig. 4E-G). Meanwhile, we also verified the single-gene diagnostic performance of DLGs in DFU, and the results showed that the respective AUC of the ROC of DLGs were 0.778 of CLU, 0.932 of ENPEP, and 0.778 of RABGEF1 (Fig. 4H). That means that DLGs have good performance as dysregulated lysosomal genes associated with DFU in predicting DFU. Additionally, we also constructed an ANN model to diagnose the onset of DFU based on the transcriptome expression pattern of DLGs. Specifically, DLGs were incorporated into an artificial neural network to predict whether a sample belonged to the Controls or DFUs (Fig. 4I). The results of the ANN prediction training set and validation set and their accuracies are shown in Table 2, in which the prediction accuracy of the training set is 86.7% and that of the validation set is 84.4%. Finally, we use ROC curves to evaluate the prediction ability of the ANN model for both the training and validation sets, where the AUC value is 0.876 (Fig. 4J) for the training set and 0.790 (Fig. 4K) for the validation set. It was demonstrated that the AUC of ROC curve is greater than 0.5, proving that the diagnostic model has some diagnostic value [40]. Overall, the ANN model is convincing as an independent diagnostic predictor of DFU.

**Table 2:** ANN diagnosis effect for the training and validation cohort

		Training cohort		Validation cohort	
		Control	DFU	Control	DFU
Prediction	Control	10	1	7	3
	DFU	3	16	2	20
	Accuracy	86.7%		84.4%	
	AUC	0.876		0.790	

### Single-gene GSEA analysis

**Fig. 5:**
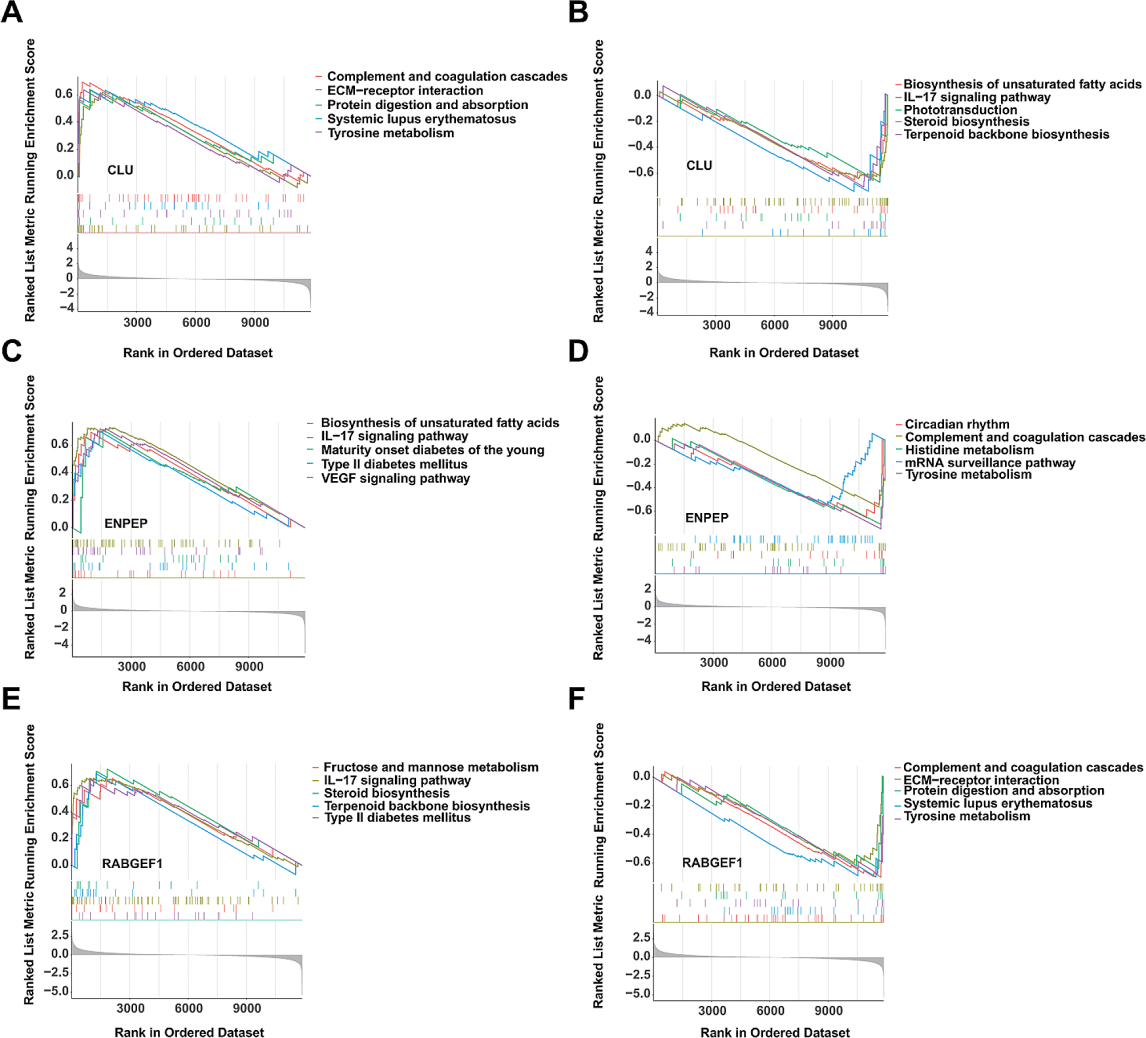
The GSEA of DLGs in DFU. (**A**-**B**) The GSEA of CLU in DFU. (**C**-**D**) The GSEA of ENPEP in DFU. (**E**-**F**). The GSEA of RABGEF1 in DFU

In order to ascertain the specific biological functions of DLGs to better understand the mechanisms of DFU. We assessed signaling pathways associated with DLGs using GSEA analysis. The top 10 signaling pathways were displayed in Fig. 5. The results showed that CLU was significantly correlated with “Complement and coagulation cascades”, “Systemic lupus erythematosus”, “Tyrosine metabolism”, “Protein digestion and absorption”, “ECM-receptor interaction”, “IL-17 signaling pathway”, “Biosynthesis of unsaturated fatty acids”, “Phototransduction”, “Terpenoid backbone biosynthesis” and “Steroid biosynthesis” (Fig. 5A-B). The expression of ENPEP significantly correlated with “IL-17 signaling pathway”, “VEGF signaling pathway”, “Maturity onset diabetes of the young”, “Type II diabetes mellitus”, “Biosynthesis of unsaturated fatty acids”, “mRNA surveillance pathway”, “Complement and coagulation cascades”, “Circadian rhythm”, “Histidine metabolism” and “Tyrosine metabolism” (Fig. 5C-D). The expression of RABGEF1 significantly correlated with “Steroid biosynthesis”, “Terpenoid backbone biosynthesis”, “IL-17 signaling pathway”, “Fructose and mannose metabolism”, “Type II diabetes mellitus”, “ECM-receptor interaction”, “Protein digestion and absorption”, “Tyrosine metabolism”, “Systemic lupus erythematosus” and “Complement and coagulation cascades” (Fig. 5E-F). Taken together, DLGs are associated with diabetes and the immune/inflammatory response signaling pathways.

### Immune cell infiltration analysis

**Fig. 6:**
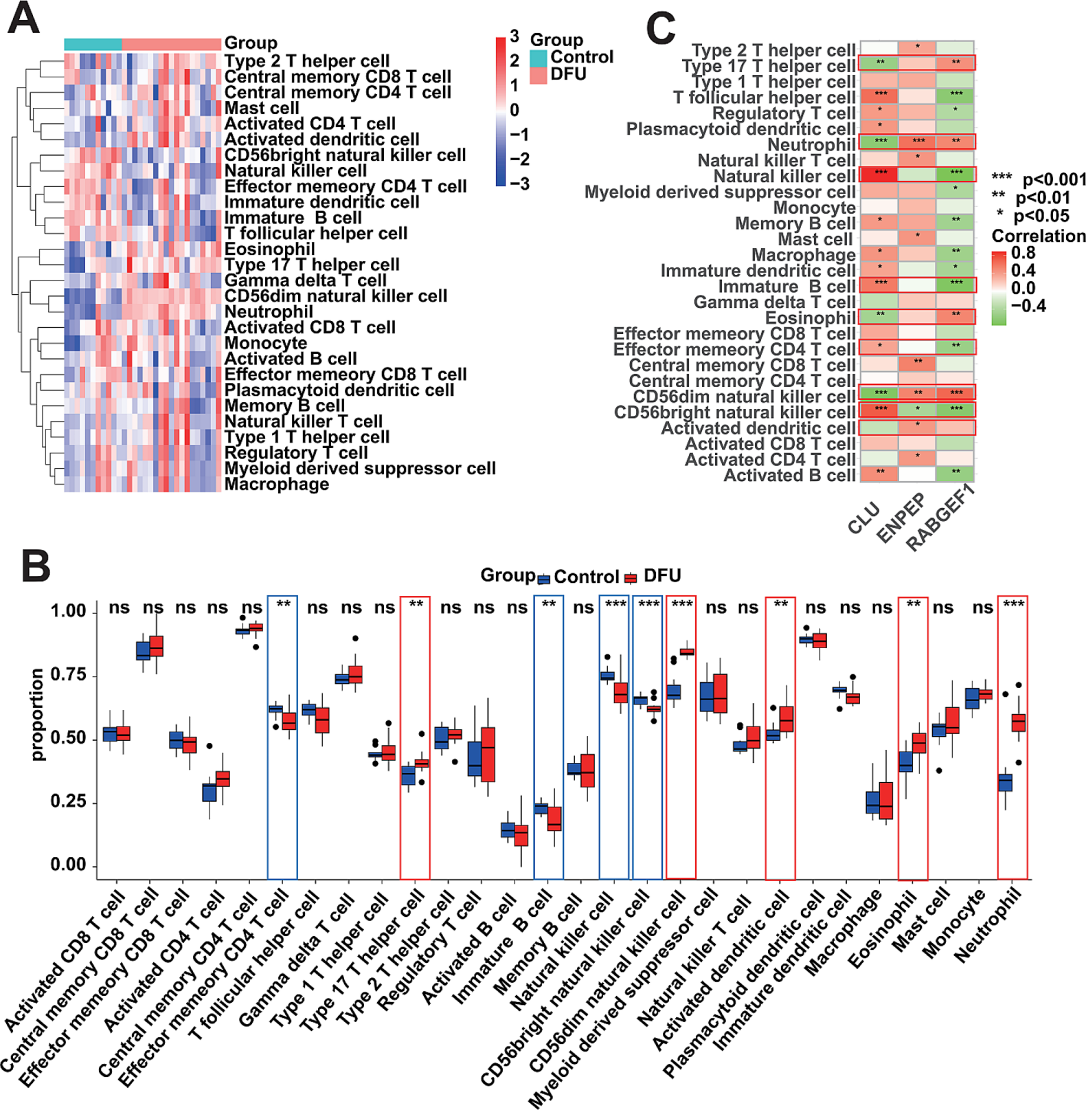
Association of immune cell infiltration abundance with DLGs. (**A**) Heatmap showing the overall landscape of immune cell abundance for DFU samples and control samples. (**B**) Box plots showing the differences in immune cell infiltration abundance between DFU samples and control samples. (**C**) Correlation analysis between the expression of DLGs and immune cell infiltration abundance. (Wilcoxon test, *p < 0.05, **p < 0.01, ***p < 0.001; ns: not significant)

Previous studies have shown that dysregulation of immune cell infiltration is also a key factor in the deterioration of DFU [41]. Thus, We performed evaluation of immunological characterization of DFU samples using the ssGSEA algorithm, that is, we calculated the of immune cells’ abundance for each sample, including control samples (Table S7). Figure 6A showed the overall immune cell infiltration, and the results indicated that there was a significant difference in immune cell infiltration between the DFUs and the Controls. Compared with controls, DFUs have lower “Effector memory CD4 T cell”, “Immature B cell”, “Natural killer cell”, “CD56bright natural killer cell”, and have higher “Type 17 T helper cell”, “CD56dim natural killer cell”, “Activated dendritic cell”, “Eosinophil” and “Neutrophil” (Fig. 6B). Taken together, we found that the abundances of the main immune cell types, such as B cells and T cells, were significantly dysregulated in the immune microenvironment of DFU. Following this, we investigated the relationship between the DLGs expression and the abundance of the nine immune cells mentioned above that are dysregulated in abundance by correlation analysis. We found significant correlations between the DLGs expression and the abundance of these 9 immune celltypes. For example, the CLU expression was positively correlated with the abundance of “Natural killer cell”, “Immature B cell” and “Effector memory CD4 T cell”, whereas the RABGEF1 expression was negatively correlated with its abundance, and then the ENPEP expression was positively correlated with the abundance of “Neutrophil”, “CD56dim natural killer cell” and “Activated dendritic cell” (Fig. 6C). The significant correlation between the DLGs expression and the immune cell abundance implies that DLGs may have a potential role in regulating the immune microenvironment of DFU.

#### Single-cell RNA-seq profiling analysis of DFU

**Fig. 7:**
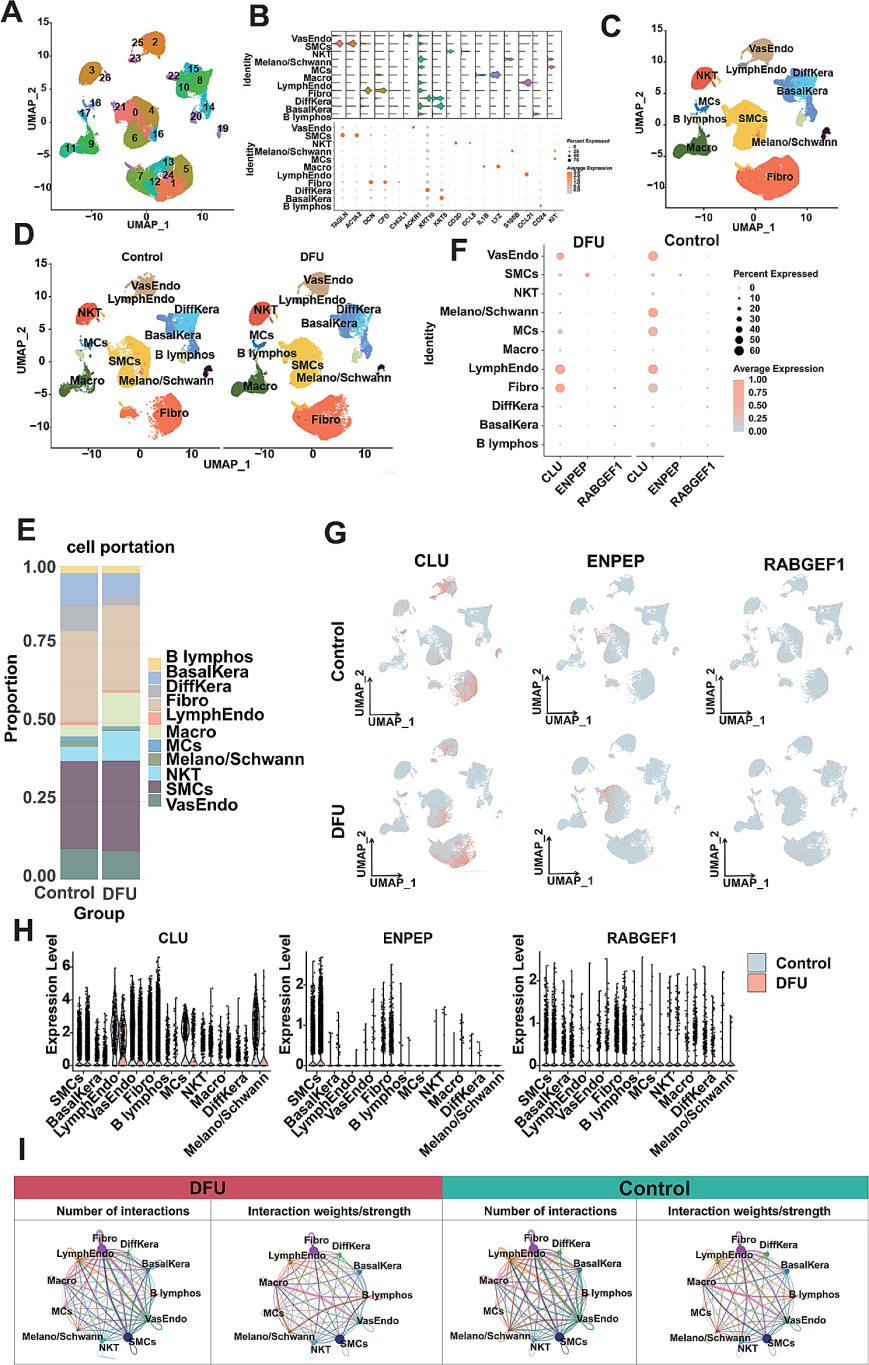
Single-cell transcriptome profiling of DFU patients. (**A**) 26 cell clusters were identified and annotated. (**B**) Expression dot plots and violin plots of known markers to support cell annotation. Namely: smooth muscle cells (SMCs: TAGLN/ACTA2), fibroblasts (Fibro: DCN/CFD/CHI3L1), vascular endothelial cells (VasEndo: ACKR1), differentiated keratinocytes (DiffKera: KRT10), basal keratinocytes (BasalKera: KRT5), NK and T cells (NKT: CD3D/CCL5), macrophages (Macro: IL1B/LYZ), melanocytes and Schwann cells (Melano/Schwann: S100B), lymphatic endothelial cells (LymphEndo: CCL21), B lymphocytes (B lymphos: CD24) and Mast cells (MCs: KIT). (**C**-**D**) Annotated single-cell transcriptome profiles. (**E**) Counting cell proportions. (**F**) Grouping expression dot plots of DLGs. (**G**) Grouping expression heatmap of DLGs. (**H**) Grouping expression violin plot of DLGs, each dot represents a cell. (**I**) Net count plots and weight/strength plots of interactions of plantar cells in DFU patients. The thicker the line indicated, the higher the number of interactions and the stronger the interaction weights/strength between two celltypes

In the present study, single-cell data from 13 DFU samples, 10 Control samples were integrated, and a total of 71,718 cells were used for further analysis after quality control. Based on the principal component analysis, we used the “RunPCA” function to reduce the dimensionality and select 18 principal components for subsequent analysis. Immediately after, we finalized 26 clusters using the UMAP algorithm (Fig. 7A), and annotated them according to known markers (Fig. 7B) [30–32]. Ultimately, 11 cell types were annotated and their single-cell profiles are shown in Fig. 7C. To investigate the cellular heterogeneity in different states, we grouped the single-cell data according to disease states (Fig. 7D). Meanwhile, we performed cell cluster statistics, which showed that BasalKera and DiffKera accounted for a higher proportion in control samples, and Macro and NKT accounted for a higher proportion in DFU samples, and this difference in proportion might exacerbate the inflammatory ulcerative status that is characteristic of DFU (Fig. 7E). Certainly, expression testing of DLGs at the single-cell level is necessary (Table S8), and the heterogeneous expression of DLGs between cells is also potentially promotive for DFU, therefore we then proceeded to explore the heterogeneous expression of DFU at the single-cell transcriptome level. The results showed that CLU showed a decreasing trend in DFU patients both in terms of expression level and expression ratio, whereas ENPEP and RABGEF1, in contrast, showed an increasing trend (Fig. 7F), and overall their expression trends were consistent with the dysregulated expression derived from bulk RNA-seq mentioned above. Immediately after that, we found that DLGs were expressed in all cell types to a certain extent by visualizing them more intuitively (Fig. 7G), but their expression was more concentrated in SMCs and Fibro cells, and the expressions and ratios of the DLGs differed between the two groups (Fig. 7H). The heterogeneous expression of DLGs at the single-cell transcriptome level was stronger evidence that lysosome-related genes can be used as diagnostic targets for DFU. Additionally, we also constructed the communication network of cells from DFU patients (Fig. 7I). The results showed that the net count and weight/intensity of interactions between cells of DFU patients were stronger than control samples (Table S9), such as the communication network of MCs, which implies that increased interactions between these cells raise the DFU risk.

### Identification and docking potential drugs that target DLGs

**Fig. 8:**
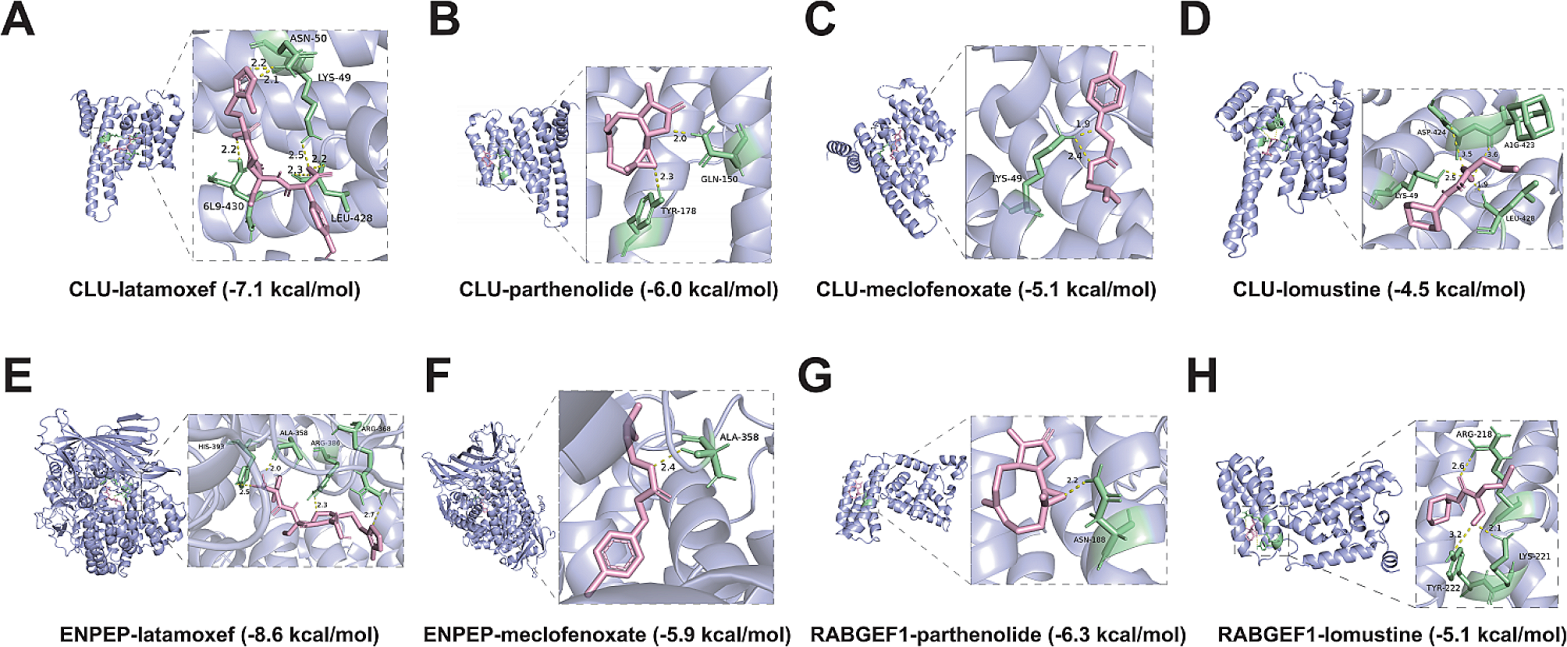
The 3D structures of complexes formed by the interaction of selected drugs with DLGs. (**A**-**D**)The structure of the complex formed by the docking of CLU with latamoxef (**A**), parthenolide (**B**), meclofenoxate (**C**) and lomustine (**D**). (**E**-**F**) The structure of the complex formed by the docking of ENPEP with latamoxef (**E**) and meclofenoxate (**F**). (**G**-**H**). The structure of the complex formed by the docking of RABGEF1 with parthenolide (**G**) and lomustine (**H**)

The previous studies have shown that the process of drug discovery begins with the identification of disease targets, and target-based drug discovery is the most common strategy for new drug development [42, 43]. The mentioned above showed that DLGs are promising as diagnostic targets for DFU, which means that we can also identify drugs that can treat or alleviate DFU by targeting DLGs. Specifically, we used the Enrichr platform (https://maayanlab.cloud/Enrichr/) for online analysis and identification. According to the DSigDB database, we identified four drugs that were able to target DLGs with p-values less than 0.05. Immediately following, we investigated the binding affinity of the selected drugs to DLGs using molecular docking techniques. The results showed that all four drugs were able to target CLU and the binding energies were relatively high, respectively, CLU-latamoxef (-7.1 kcal/mol), CLU-parthenolide (-6.0 kcal/mol), CLU-meclofenoxate (-5.1 kcal/mol) and CLU- lomustine (-4.5 kcal/mol) (Fig. 8A-D). Subsequently, latamoxef and meclofenoxate were both able to target ENPEP, respectively ENPEP-latamoxef (-8.6 kcal/mol) and ENPEP-meclofenoxate (-5.9 kcal/mol) (Fig. 8E-F). Additionally, parthenolide and lomustine were also able to target RABGEF1, respectively RABGEF1-parthenolide (-6.3 kcal/mol) and RABGEF1-lomustine (-5.1 kcal/mol) (Fig. 8G-H). Taken together, the docking results suggest that these potentially targeted drugs may influence the development of DFU by interacting with DLGs to treat or alleviate the DFU symptoms.

## Discussion

DFU is a serious complication of diabetes mellitus [44], which has become a global health problem [45]. According to current statistics, approximately 80% of lower limb amputation patients have diabetic foot before surgery [46]. Although some technologies have been developed to treat DFU, the current clinical interventions and efficacy for DFU fail to reach expectations [47, 48]. The high disability and mortality rates of DFU not only impose serious financial, physical and mental burdens on patients, but also cause a heavy burden on healthcare and nursing care, thus it is urgent to identify novel molecular therapeutic targets to help improve patient prognosis and reduce disability [3]. Previous studies have shown that lysosomal abnormalities lead to various autoimmune and metabolic diseases including diabetes, but DFU, as the most common and serious complication of diabetes, lysosomal abnormalities also contribute to DFU development [17]. In addition, lysosomes as novel targets for autoimmune, metabolic and renal diseases have been frequently reported in recent years [49]. Thus, in the present study, we explored the potential of lysosomes as biomarkers for DFU, aiming to contribute to the diagnosis and treatment of DFU.

In the present study, we comprehensively analyzed DFUs based on bulk RNA-seq and scRNA-seq data by various bioinformatic and machine learning algorithms. Specifically, we first identified DEGs associated with DFU by differential analysis and WGCNA analysis, and isolated lysosome genes from DEGs using lysosome genes annotated in AmiGO2 for lysosomal genes with potential regulation in DFU. Notably, previous analyses were analyzed and identified based on transcriptome expression patterns only, without taking into account the interactions between the genes themselves, so we utilized PPI analysis to ensure the accuracy of our results on a multidimensional level. Subsequently, we performed enrichment analysis on these genes to validate their reliability, and the results showed that they were significantly associated with lysosomes, substance transport across membranes, and immune/inflammatory responses, which implies a certain degree of reliability of our results. To identify DLGs in DFUs, we applied three machine learning algorithms, and the results showed that CLU, ENPEP and RABGEF1 were indicated by all machine learning algorithms, implying their potential regulatory role in the DFU development. It’s worth mentioning that these genes have been shown to be used to treat other diseases as well, for example, CLU can serve as a marker and mediator of chemoresistance in colorectal cancer [50]. Iron deficiency in liver tissue downregulates ENPEP, promotes angiogenesis in liver tumors and is associated with poor prognosis in hepatocellular carcinoma patients [51]. Epithelial RABGEF1 deficiency promotes intestinal inflammation by dysregulating intrinsic MYD88-dependent innate signaling [52].

It is possible that DLGs may be involved mechanistically in the occurrence and development of DFUs, and thus may also be a potential diagnostic target for DFUs. Certainly, validation of the diagnostic performance of DLGs for DFU requires artificial neural network modeling [53]. As one of the main types of artificial intelligence, ANN has been widely used in clinical medicine for diagnosis and treatment by virtue of its better algorithms and accuracy [54, 55]. However, there are still some deficiencies in the current clinical diagnosis of DFU, especially the diagnosis based on the molecular level is not yet complete, and very often relies on the clinical experience of clinicians [56]. Therefore, we built an ANN model to predict whether the samples in the present study are control or DFU based on DLGs transcriptome patterns, in which more than 80% prediction accuracy was achieved for both the training and validation sets. Meanwhile, we evaluated the prediction ability of the ANN model for the training and validation sets with ROC curves, in which the AUC value of the training set was 0.876 and the AUC value of the validation set was 0.790, which suggests that there is potential for an ANN built based on the DLGs transcriptome pattern to be an independent diagnostic predictor of DFU.

Dysregulation of immune cell infiltration is also a key factor in the deterioration of DFU [41]. In molecular terms, the wound healing process occurs after the breakdown of the skin’s protective barrier. Wound healing is a regulated and highly complex biological process. It is reported that immune/inflammatory regulation is crucial in the wound healing process [57]. To investigate the role of immune/inflammatory cells in DFU wound deterioration, we performed a comprehensive assessment of the immune microenvironment of DFUs and presented different cellular components in the immune microenvironment between DFUs and Controls. Our study demonstrated the high abundance of eosinophils and neutrophils in DFUs. It has been previously shown that neutrophils are the first inflammatory leukocytes to migrate to the wound site, and that they are capable of eliminating invading pathogens and initiating inflammatory and non-inflammatory responses through various mechanisms [58], implying that the high abundance of neutrophils in DFUs may worsen the symptoms of DFUs through excessive inflammatory responses. Additionally, we found a significant abundance decrease of effector memory CD4 T cells, immature B cells, and natural killer cells in DFUs. The significant decrease in the abundance of B/T cells and natural killer cells, the mainstay of defense against exogenous pathogenic microorganisms, also increased the risk of worsening of DFU wound infections.

The technology of single-cell RNA sequencing presents itself as an advanced method for revealing the complexity and heterogeneity of RNA transcripts within a single cell, as well as the composition and function of different cell types within a tissue [59]. Relying on the advantages of single-cell RNA sequencing technology in studying tissue heterogeneity, we also utilized this technology to perform single-cell profiling of DFUs. Our study showed that DLGs are also heterogeneously expressed among different cell types, which also provides stronger evidence that lysosome-related genes can be used as diagnostic targets for DFU. In addition, we found that the percentage of macrophages in DFUs was significantly higher compared with controls. It has been previously shown that macrophages play a role in hemostasis and that pro-inflammatory macrophages infiltrate and remove dead cells and bacteria from the wound site after injury [60]. During the wound healing phase, macrophage polarization stimulates the movement and growth of fibroblasts, endothelial cells, and keratinocytes, which accelerates repair of the epidermis, dermis, and vascular system [57]. Additionally, it has been shown that decreased innate immunoregulatory function exacerbates macrophage polarization imbalance as well as decreased wound healing [61], which is also consistent with the results of the immune infiltration analyses mentioned above, suggesting that a significant down-regulation of the abundance of dominant immune cells in DFUs, including B and T cells, leads to a decrease in wound healing capacity.

DLGs have not only been validated from bulk RNA-seq and scRNA-seq, but also play a key role in regulating the immune and inflammatory states of DFU, which means that DLGs are promising targets for the diagnosis and treatment of DFU, and it is also possible to identify drugs to treat DFU by targeting DLGs. Previous studies have shown that the process of drug discovery begins with the identification of disease targets, and target-based drug discovery is the most common strategy for new drug development [42, 43]. Thus, targeting DLGs may provide a new effective therapeutic approach for DFU treatment. In the present study, we identified four potential drugs targeting DLGs using the Enrichr platform: latamoxef, parthenolide, meclofenoxate, and lomustine. The identified targeted drugs have also been demonstrated to treat immune/inflammatory diseases, including cancer, in previous study reports. For example, Latamoxef was able to largely treat febrile neutropenic patients [62]. Parthenolide has potential therapeutic effects in luminal A breast cancer [63]. Meclofenoxate is effective in the treatment of neuroleptic-induced dyskinesia [64], as well as alcoholism and dementia [65]. Lomustine has shown favorable results in the treatment of glioblastoma [66, 67]. Subsequently, we used molecular docking technique to deeply investigate the binding affinity between the mentioned above drugs and their DLGs. The results indicated that the above identified targeting drugs (latamoxef, parthenolide, meclofenoxate and lomustine) may be promising therapeutic drugs against DFU, but their therapeutic effects on DFU still need to be proved by numerous cellular and animal experiments.

Certainly, we must admit that there are limitations in the present study. First, our analyses were based only on samples from the training cohort, and although the reliability of the analyzed results was also validated in a dataset independent of the present study, they are still at an early stage and larger clinical samples are needed to corroborate them. Secondly, our work requires further validation by numerous in vivo and in vitro experiments, which will be the focus of our future research endeavors.

## Conclusions

In summary, we identified DLGs for DFU using three machine learning algorithms and validated the diagnostic performance of these DLGs in an independent dataset. Meanwhile, we built a novel ANN model based on the transcriptomic patterns of DLGs that is promising for improving the clinical diagnosis of DFUs. Additionally, we investigated the correlation between the DLGs expression and the immune cell abundance in DFU, and demonstrated the heterogeneous expression of DLGs among different cells using single-cell analysis techniques. Finally, we identified promising therapeutic drugs against DFU by targeting DLGs using the Enrichr platform and molecular docking technology. These findings contribute to the understanding of the pathogenesis of DFU, help to improve the prognosis of DFU patients and reduce the disability rate, as well as provide new perspectives on the diagnosis and therapeutic strategies of DFU.

### Electronic supplementary material

Below is the link to the electronic supplementary material.

Supplementary Material 1

Supplementary Material 2

## Data Availability

Datasets related to this article are from public database (GSE165816, GSE134431, GSE80178, GSE7014 and GSE68183). All data generated or analyzed during this study are included in this article/Additional files, further included can be directed to the corresponding author.

## Abbreviations

DFU: Diabetic Foot Ulcers
ANN: Artificial Neural Network
PCA: Principal Component Analysis
DEGs: Dysregulated Expressed Genes
TOM: Topological Overlap Matrix
PPI: Protein-Protein Interactions
GO: Gene Ontology
CLU: Clusterin
ENPEP: Glutamyl Aminopeptidase
RABGEF1: RAB Guanine Nucleotide Exchange Factor 1
DLGs: Hub Lysosome-Related Genes of DFU
ROC: Receiver Operating Characteristic
AUC: Area Under Roc Curve
GSEA: Gene Set Enrichment Analysis
ssGSEA: single-sample Gene Set Enrichment Analysis
PCs: Principal Components
SMCs: Smooth Muscle Cells
Fibro: Fibroblasts
VasEndo: Vascular Endothelial Cells
DiffKera: Differentiated Keratinocytes
BasalKera: Basal Keratinocytes
NKT: NK and T Cells
Macro: Macrophages
Melano/Schwann: Melanocytes and Schwann Cells
LymphEndo: Lymphatic Endothelial Cells
B lymphos: B lymphocytes
MCs: Mast Cells
MDM: Molecular Docking Method
SVM-RFE: Support Vector Machine Recursive Feature Elimination
LASSO: Least Absolute Shrinkage and Selection Operator
RF: Random Forest
ABI: Ankle-Brachial Index
NCV: Nerve Conduction Velocity
AGE: Advanced Glycation End-Product
