# Supplementary material for: Development of novel lysosome-related signatures and their potential target drugs based on bulk RNA-seq and scRNA-seq for diabetic foot ulcers
Source: Hum Genomics. 2024 Jun 11;18:62. doi: 10.1186/s40246-024-00629-1 (PMC11165785; doi:10.1186/s40246-024-00629-1)
Supplement: Supplementary file 2 — Supplementary Material 2 [file 40246_2024_629_MOESM2_ESM.docx]

**
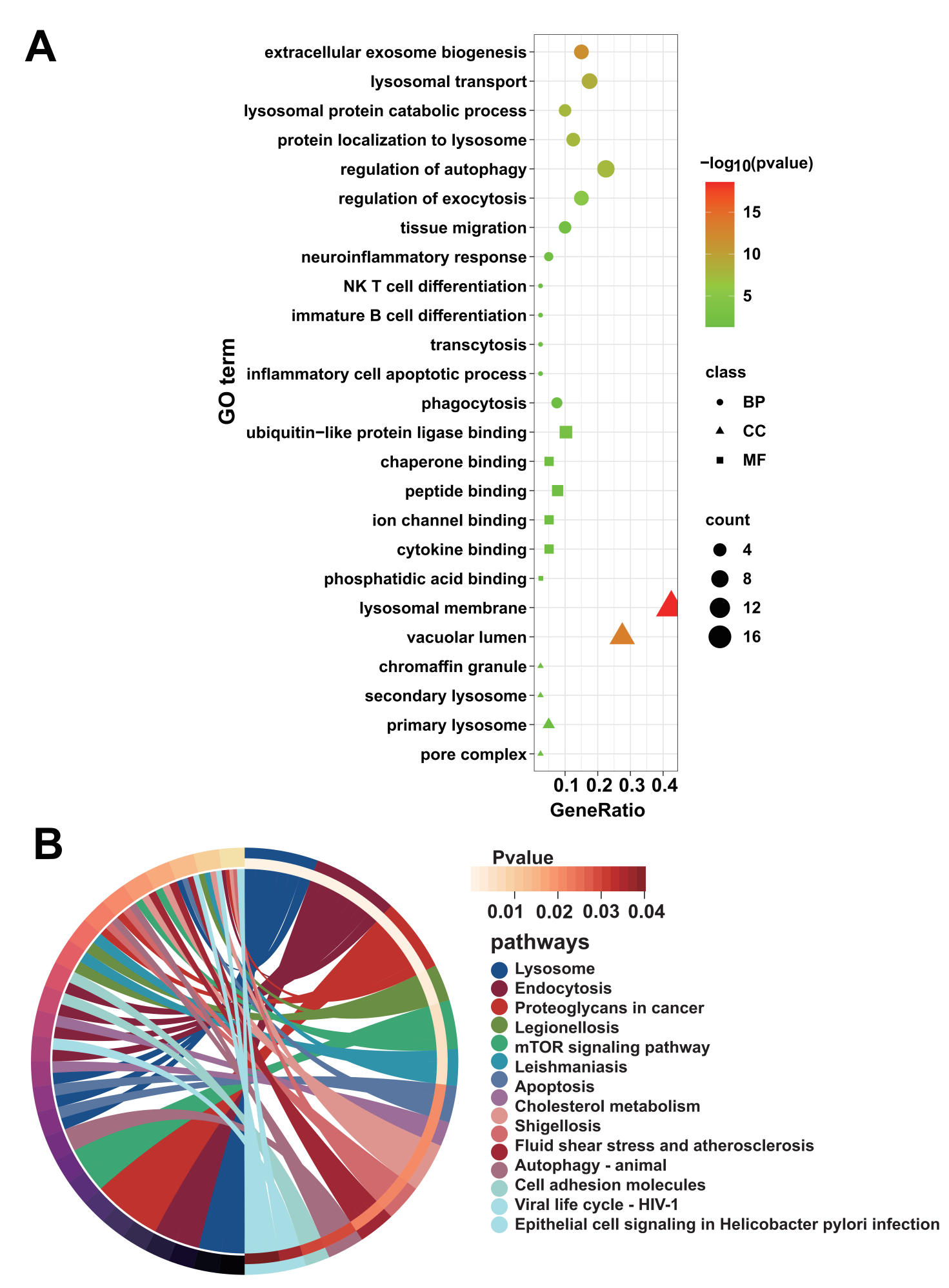
**

**Figure S1. Functional enrichment analysis of top 40 scoring genes. (A)** GO enrichment resultsof the top 40 scoring genes. **(B)** KEGG enrichment results of the top 40 scoring genes.
